# Père David’s deer gut microbiome changes across captive and translocated populations: Implications for conservation

**DOI:** 10.1111/eva.12743

**Published:** 2019-01-03

**Authors:** Lei Wang, Jingjing Ding, Zhisong Yang, Hua Chen, Ran Yao, Qiang Dai, Yuhua Ding, Lifeng Zhu

**Affiliations:** ^1^ Nanjing Normal University College of Life Sciences Nanjing China; ^2^ Jiangsu Academy of Forestry Nanjing China; ^3^ Key Laboratory of Southwest China Wildlife Resources Conservation (Ministry of Education) China West Normal University Nanchong China; ^4^ Shanghai Biozeron Bioinformatics Center Shanghai China; ^5^ Chengdu Institute of Biology, Chinese Academy of Sciences Chengdu China; ^6^ Jiangsu Dafeng Milu National Nature Reserve Dafeng China

**Keywords:** coevolution, conservation, gut microbiome, Père David’s deer, sodium transport system, translocated populations

## Abstract

The gut microbial composition and function are shaped by different factors (e.g., host diet and phylogeny). Gut microbes play an important role in host nutrition and development. The gut microbiome may be used to evaluate the host potential environmental adaptation. In this study, we focused on the coevolution of the gut microbiome of captive and translocated Père David's deer populations (*Elaphurus davidianus*; Chinese: Père David's deer). To address this, we used several different macro‐ and micro‐ecological approaches (landscape ecology, nutritional methods, microscopy, isotopic analysis, and metagenomics). In this long‐term study (2011–2014), we observed some dissimilarities in gut microbiome community and function between the captive and wild/translocated Dafeng Père David's deer populations. These differences might link microbiome composition with deer diet within a given season. The proportion of genes coding for putative enzymes (endoglucanase, beta‐glucosidase, and cellulose 1,4‐beta‐cellobiosidase) involved in cellulose digestion in the gut microbiome of the captive populations was higher than that of the translocated population, possibly because of the high proportion of cellulose, hemicellulose, and lignin in the plants most consumed by the captive populations. However, the two enzymes (natA and natB) involved in sodium transport system were enriched in the gut microbiome in translocated population, possibly because of their high salt diet (e.g., *Spartina alterniflora*). Thus, our results suggested that Père David's deer gut microorganisms potentially coevolved with host diet, and reflected the local adaptation of translocated population in the new environment (e.g., new dietary plants: *Spartina alterniflora*). A current problem for Père David's deer conservation is the saturation of captive populations. Given that the putative evolutionary adaptation of Père David's deer gut microbiome and its possible applications in conservation, the large area of wetlands along the Yellow Sea dominated by *S. alterniflora* might be the major translocation region in the future.

## 1 INTRODUCTION

Translocation (e.g., introduction and reintroduction) is an effective conservation management strategy that decreases extinction risk by increasing species ranges, augmenting critical populations, and establishing new populations (Rout, Hauser, & Possingham, 2005). Some translocated individuals may originate from captive environments. However, wild environments may differ from captive environment in many ways, including diet. Wild animals predominantly obtain nutritional energy from wild ecosystems. One of the most important connections between mammals and their food is provided by symbiotic gut microorganisms, which play an important role in host nutrition and development (Ley et al., 2008). Several recent evolutionary ecology studies have investigated the relationship between the diet of the mammal host and the symbiotic gut microbial community (Faith, McNulty, Rey, & Gordon, 2011). Thus, the gut microbiome may be used to evaluate the host potential environmental adaptation (e.g., diet). Furthermore, given the putative connection between conservation and microbiomes (Bahrndorff, Alemu, Alemneh, & Nielsen, 2016; Metcalf et al., 2017; O'Doherty et al., 2014; Redford, Segre, Salafsky, Martinez, & del Rio, and D McAloose., 2012; Stumpf et al., 2016), changes in the gut microbiome dynamics of translocated populations might an unavoidable consequence of translocation. However, these dynamics have yet to be investigated.

**Figure 1:**
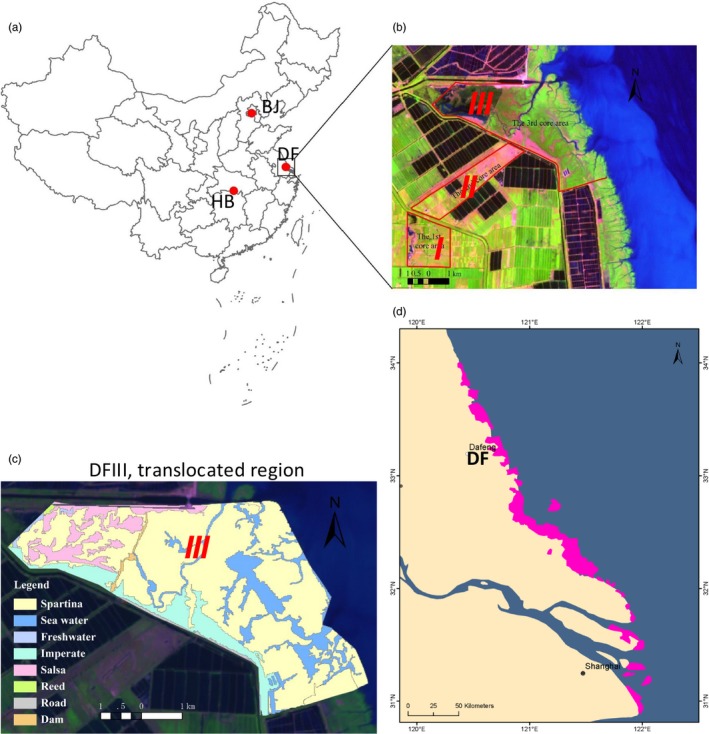
The study area. (a) The main Père David's deer populations in China: Dafeng Natural Reserve (DF), Jiangsu; Hubei Shishou Natural Reserve (HB), Hubei; and Beijing Nanhaizi Natural Reserve (BJ), Beijing. (b) The satellite map of DF (composed of three core areas: DFI, DFII, and DFIII), which holds the largest Père David's deer population in the world. (c) The wild habitat (DFIII) in 2013. Shown is the distribution of plants, including Spartina (SAL: *Spartina alterniflora*.), Imperate (ICY: *Imperata cylindrica var. major*), Salsa (SGL: *Suaeda glauca*), Reed (PAU: *Phragmites australis*), sea water, and roads. (d) The distribution range of SAL (purple regions in Jiangsu and Shanghai provinces were sketched using the record from our previous surveys)

Père David's deer (*Elaphurus davidianus*), endemic to China, were once widely distributed in East Asia, although primarily in China. Père David's deer was first introduced to the west in 1866 by Armand David (Père David) (Cao, 1985). This species became extinct in China in the early 20th century. Fortunately, between 1894 and 1901, Herbrand Arthur Russell, the 11th Duke of Bedford, acquired the few remaining Père David's deer (18 individuals) from European zoos and nurtured them at Woburn Abbey, England (Cao, 1985). All currently living Père David's deer stem from this herd (Cao, 1985). In the mid‐1980s, 77 deer were reintroduced into China in captivity; populations were later established in Beijing, Dafeng, and Hubei Shishou (Figure 1a). Currently, the Dafeng Nature Reserve (DF) harbors the largest population of Père David's deer in the world (~2,800 individuals) (Figure 1a; Ding, 2017).

Chinese Père David's deer live in three core areas (Ding, Ren, Wen, Li, & Chang, 2014). Core Areas I (DFI) and II (DFII) are captive environments (Figure 1b): Deer in these areas are fenced in and eat naturally occurring plants. However, due to overgrazing and the periods when grass is withered (November to April), the naturally occurring plants are insufficient, and the diets of the captive deer are supplemented with human‐provided grains: wheat bran, barley, corn, soybean, and soybean straw fibers. The most common food plants in DFI and DFII are *Pennisetum alopecuroides* (PAL), *Imperata cylindrica var. major* (ICY), and *Phragmites australis* (PAU). DFIII is a wild habitat; 53 deer have been translocated into this area since 1998 (Figure 1b; Ding, Zhu, & Ren, 2006). At present, 215 Père David's deer inhabit DFIII (Ding et al., 2014). The most abundant potential food plants in DFIII are high salty *Spartina alterniflora* (SAL), PAU, *Suaeda glauca* (SGL), PAL, and ICY (Figure 1c). The concentration of salty of the Père David's deer dietary plants in DFIII is significantly high than that of dietary plants in DFI and DFII (Zhu, Deng, et al., 2018).

American Bison (*Bison bison*) adjust their diet continuously over the growing season, possibly dictated by the seasonal availability of high‐protein plant species (Bergmann, Craine, Robeson, & II, and Noah Fierer., 2015). The diet of bison during summer and fall has higher caloric and protein (Craine, Towne, Tolleson, & Nippert, 2013). The seasonal shift in diet is associated with a significant increasing in the abundance of *Tenericutes* from spring to summer in the bison gut microbiome (Bergmann et al., 2015). Some *Tenericutes* specifically ferment simple sugars (Manurung, Boye, & Mølbak, 2012). Differences in gut microbial communities have been identified between the Beijing and Shishou Père David's deer populations using 16S RNA gene sequences; these differences be associated with the differing abundances of the available plant species (Meishan et al., 2018). Père David's deer diets require further detailed investigation to evaluate the possible effects of diet on gut microbe composition (Meishan et al., 2018). Further knowledge of gut microbiome function will increase our understanding of the relationship between diet and gut microbiome.

In this study, we therefore aimed to determine how Père David's deer gut microorganisms are influenced by different food sources in both natural and captive environments at a fine scale. We used different approaches (landscape ecology, nutritional analysis, microscopy, isotopic analysis, and metagenomics) to characterize the regional dynamics of gut microorganism composition and function between translocated and captive populations (two captive, DFI and DFII; one wild, DFIII) with respect to different habitat. We then aimed to assess the relevance of our results for Père David's deer conservation in the future.

## 2 MATERIALS AND METHODS

### 2.1 Diet analysis

#### 2.1.1 Field observations

Although the feeding behaviors of captive Père David's deer can be observed in close proximity, those of wild/translocated Père David's deer cannot because of the inaccessibility and topography of Dafeng Père David's deer National Nature Reserve, as well as the specific instrumentation requirements associated with observation. The staple foods of captive Père David's deer in DFI and DFII were ascertained by field observation, and the feeding times were recorded every summer and winter from 2011 to 2014. In DFIII, we tracked the traces of Père David's deer (e.g., footprints, bedding sites, and feces) between 2011 and 2014. Food species, feeding sites, and observation times were recorded. Evidence of forage feeding was preliminarily determined by monitoring what the deer had left behind. This approach, in combination with the guidance of the Dafeng Père David's deer National Nature Reserve staff and the data of previous researchers, allowed us to determine the staple food requirements of the Père David's deer in DFIII.

#### 2.1.2 Fecal microhistology

Plant species were investigated and related to season‐specific data. Sample lines were run following the advice of the Dafeng Père David's deer National Nature Reserve staff, where evidence of feeding and foraging were identified. We collected 21 species of plants (belonging to seven families and 21 genera), which had a high probability of being foraged by the deer in DFIII, from Dafeng Père David's deer National Nature Reserve between 2011 and 2014. Père David's deer fecal samples (*n* = 255 dung piles) were collected in November 2011, December 2011, January 2012, February 2012, March 2012, June 2012, December 2012, August 2013, September 2014, and November 2014. Three fecal pellets from each dung pile were mixed to form 17 composite samples over each half‐year period. The times associated with sampling were divided into two seasons: summer (from June to September) and winter (from November to March).

All plant materials were oven‐dried at 80°C for 72 hr and then ground over an 80‐mesh screen. Ground plant matter was placed in a valve bag in an envelope. We weighted ~1 g of each sample in a shaded petri dish containing sodium hypochlorite solution. To evenly distribute material, samples were stirred hourly with a dissecting needle. After 3–5 hr, we prepared temporary slides to determine whether cells were clear. The required time for sodium hypochlorite incubation depends on temperature: The higher the temperature, the shorter the treatment time. Once cells were clear, each sample was washed over a 200‐mesh screen. After 2 min, samples were moved to new petri dishes and stained with one to two drops of safranine for ~30 min. Sample was then washed again to remove excess dye. After 2 min, several epidermal fragments were placed on the slide in a drop of distilled water. The water was then absorbed using filter paper. Small amounts of glycerin were added until the fragment samples were fully covered, and the mixture was stirred, covered with a coverslip, and sealed with neutral gum (Wang & Wang, 2011). Microscopic slides of fecal samples were prepared in an identical manner. Three slides were prepared from each composite sample.

Slides were examined under a Nikon H550L microscope at 100× or 200× magnification and imaged with a DS‐FIZ K12338 digital imaging system at 200 × magnification. For each fecal sample slide, ten microscopic fields were examined. Images were captured and identified similarly to the reference plants. All identifiable fragments were counted. Epidermal fragments from fecal samples were identified based on morphological differences among cells, including cell size, cell shape, trichomes presence, trichome size, and stomatal apparatus density. The frequency conversion technique (Sparks & Malechek, 1968) was used. The frequency percentage can be converted to the density of recognized plant particles (*D*), and the density of particles can subsequently be converted to relative density (RD) as.

$$RD=\left(\mathrm{cuticlefragmentdensityforeachidentifiedplantspecies}\right)/\left(\mathrm{totalcuticlefragmentdensitiesacrossallidentifiedplantspecies}\right)\times 100\%.$$

RD also represented the proportion of the plant composed of dry weight material. This value can be used to estimate the actual proportion of each plant in the food samples.

### 2.2 Stable carbon and nitrogen isotopes

#### 2.2.1 Sample collection

Samples of 89 plants were collected from the Dafeng Père David's deer National Nature Reserve in August 2013. From DFI, we collected seven PAL samples; from DFII, we collected nine ICY samples and four PAL samples; from DFIII, we collected 40 SAL samples, eight *Erigeron annuus* samples, 12 PAU samples, four SGL samples, and five *Tamarix chinensis* samples. The upper leaves and stems (which sometimes contained a limited number of leaves) were collected from SAL, *Erigeron annuus,* and PAU.

We collected 438 fecal samples: 34 from DFI in the summer (2013 and 2014), 40 from DFI in the winter (2011 and 2014), 36 from DFII in the summer (2013 and 2014), 48 from DFII in the winder (2011 and 2014), 95 from DFIII in the summer (2012–2014), and 113 from DFIII in the winter (2011, 2012, and 2014).

#### 2.2.2 Sample preparation

We used a Delta Plus Advantage Isotope Ratio Mass Spectrometer at the Nanjing Institute of Geography and Limnology, Chinese Academy of Sciences (Nanjing China), for isotopic analyses. Standard reference materials were used: carbon from the Peedee limestone (PDB) and nitrogen gas in the atmosphere (Peterson & Fry, 1987). Fecal samples were oven‐dried at 60°C for 48 hr. After powdering, the material was weighed in a tinfoil capsule using an electronic balance (accurate to 0.000001 g). We weighed 0.1 mg and 2.0 mg of powder to measure the carbon and nitrogen isotopes, respectively.

#### 2.2.3 Statistical analyses

We expressed the ^13^C/^12^C and ^15^N/^14^N ratios in delta (δ^13^C, δ^15^N) notation in parts per thousand (‰) relative to the PDB standard as follows:

$$\delta^{13}C=\left[{\left({}^{13}C{/}^{12}C\right)}_{\mathrm{sample}}/{\left({}^{13}C{/}^{12}C\right)}_{\mathrm{standard}}-1\right]\times 1000\;\;\mathrm{and}$$

$$\delta^{15}N=\left[{\left({}^{15}N{/}^{14}N\right)}_{\mathrm{sample}}/{\left({}^{15}N{/}^{12}N\right)}_{\mathrm{standard}}-1\right]\times 1000$$

We used the Shapiro–Wilk test to determine whether our data were normally distributed (Park, 2008) and used Levene's test to determine homogeneity of variance. Nonparametric statistical methods were used because neither the raw nor the converted data were parametric. The fecal δ^13^C values from two independent samples were compared, and the Mann–Whitney *U* test was used to compare the food intake in captive Père David's deer to that of wild Père David's deer. Differences in fecal δ^15^N values from different regions were analyzed using k‐independent samples and the Kruskal–Wallis *H* test. Data were analyzed using Microsoft Excel 2010 and IBM SPSS Statistics 20.0. We considered *p* < 0.05 statistically significant.

### 2.3 Nutritional analysis of the staple foods of Père David's deer

#### 2.3.1 Sample collection

The sample collection method used here was identical to that of the isotope analysis.

#### 2.3.2 Sample preparation and analysis

Fresh plants were weighed and then oven‐dried at 80°C for 72 hr. After recording the dry weight, plant materials were ground over an 80‐mesh screen and subsequently placed into a valve bag in an envelope. We weighted ~1–2 g of powder for the plant nutrition analysis. Several factors relevant to nutritional content, including water, crude protein, crude fat, soluble saccharides, hemicellulose, cellulose, and lignin, were assessed. Water content was measured with the weigh method; crude protein was measured with the Kjeldahl nitrogen method; crude fat was measured with the Soxhlet extraction method; soluble saccharides were measured with the anthrone colorimetric method; and salinity was measured using conductivity (similar to the test for soil salinity). Hemicellulose, cellulose, and lignin composition were determined using the Van Soest method (Van Soest, Robertson, & Lewis, 1991; Zhang, Deng, Zhuang, & Lin, 2003). All data were shown as percentages. Because of the small sample sizes, data normality was determined using the Shapiro–Wilk test (Park, 2008). Homogeneity of variance was measured using Levene's test. Nonparametric statistical methods were used when the raw and converted data were not parametric. Differences in nutritional content between two independent samples were identified using the Mann–Whitney *U* test. In this way, differences between the staple foods of the deer from different regions were assessed. Data were analyzed using Microsoft Excel 2010 and IBM SPSS Statistics 20.0. We considered *p* < 0.05 statistically significant.

### 2.4 Gut microbial community analysis

#### 2.4.1 Sample collection

Père David's deer feeding was observed daily. After the herd left the feeding locations, fresh fecal samples were collected. Fresh fecal samples were collected in Dafeng Père David's deer National Preserve from 2011 to 2014. The fresh feces were placed in an icebox (−20°C) and then shipped to the laboratory. The samples were stored and then used for isotopic and gut microbial analysis. Microbial data were generated for 315 samples. Most of the fresh samples were used to generate both isotopic and gut microbial data. Microbial DNA was extracted from all 315 samples using the Qiagen Stool DNA Kit (USA). The V4–V5 region of the bacterial 16S ribosomal RNA gene was amplified using the primers 515F (5′‐barcode‐GTGCCAGCMGCCGCGG‐3′) and 907R (5′‐CCGTCAATTCMTTTRAGTTT‐3′), where the barcode was an eight‐base sequence unique to each sample. Library construction (2 × 250 bp) and sequencing were performed on an Illumina MiSeq platform by Shanghai BIOZERON Co., Ltd. (Shanghai, China) according to standard protocols.

#### 2.4.2 Processing of sequence data

After quality‐filtering using QIIME1.9.1 (Caporaso et al., 2010), we identified operation taxonomic units (OTUs) in the clean dataset using UPARSE 7.1 (http://drive5.com/uparse/; with a 97% similarity cutoff). Chimeric sequences were identified and removed using UCHIME. Each 16S rRNA gene sequence was assigned to bacterial group with the RDP Classifier (http://rdp.cme.msu.edu/; against the silva (SSU123)) 16S rRNA database, using a confidence threshold of 70% (Amato et al., 2013).

#### 2.4.3 Data analysis for the central hypothesis

We treated the two sampling seasons (summer and winter) as separate replicates to evaluate the relationship between gut microbiome composition (alpha and beta diversity) and diet within each sampling season. The alpha diversity (i.e., Shannon index and phylogenetic diversity) for each fecal sample was calculated with QIIME (Caporaso et al., 2010). We identified significant differences in the abundance of bacterial taxa among the three core areas using the linear discriminant analysis effect size method (Lefse; Segata et al., 2011). To identify dissimilarities in community composition, we performed a principal coordinates analysis (PCoA) analysis in QIIME (Caporaso et al., 2010). Supervised learning analyses (random forests) were performed in QIIME to determine whether sampling season or core area (location) could be used to differentiate samples based on microbial composition (OTUs; Breiman, 2001; Caporaso et al., 2010; Knights, Costello, & Knight, 2011). This analysis calculated the ratio of baseline error to the estimated generalization error for the random forests classifier. A reasonably good classification should produce a ratio ≥2 (Hale et al., 2018), indicating that the random forests classifier was at least twice as accurate as random guessing. We expected that this random forests classifier would perform better at discriminating captive deer from wild deer (i.e., DFI + II vs. DFIII) than differentiating the two collection seasons across group type (i.e., summer captive + summer wild vs. winter captive + winter wild), because of the similar diets of from DFI and DFII. Moreover, to evaluate the effect of diet across captive and translocated populations, we performed one‐way PERMANOVA on Bray–Curtis dissimilarities in PAST3 (Hammer, Harper, & Ryan, 2001) to test the microbial community composition.

### 2.5 Metagenomic analysis

We analyzed the metagenomic data of 24 Père David's deer fecal samples (Zhu, Yang, et al., 2018). STAMP (Parks, Tyson, Hugenholtz, & Beiko, 2014) was used to identify differences in significantly enriched KEGG (Kyoto Encyclopedia of Genes and Genomes) pathways among the three core habitats (DFI, DFII, and DFIII). Taxonomic classifications of predicted gene sequences were determined using MEGAN5 (Huson, Auch, Qi, & Schuster, 2007).

### 2.6 DFIII habitat analysis

Habitat was evaluated based on the distribution of various plant taxa, as determined from satellite images taken in 2013. These images covered the entire Père David's deer distribution range in DFIII. Using the maximum‐likelihood classification algorithm in supervised classification, forest cover was identified using ERDAS IMAGE 8.7 (Leica Geosystems GIS and Mapping 2003). We computed related landscape indices to compare the habitat changes between different time periods using FRAGSTATS 3.3 (McGarigal & Marks, 1995).

## 3 RESULTS

### 3.1 Diets of captive (DFI and DFII) and translocated (DFIII) Père David's deer

Fecal microhistology indicated that PAL (C4) and ICY (C4) were the staple components of the diets of Père David's deer in DFI and DFII during the summer, with these species comprising 77.09% of the total diet in DFI and 81.45% in DFII (Table 1). These results suggested that the diets of the deer from DFI and DFII were similar in the summer. However, during the winter, the staple foods of the Père David's deer in DFI and DFII were predominantly the dicotyledonous and gramineous plants (e.g., wheat bran, barley, soybean, and corn straw fibers) provided by humans. This reflected the difference in staple foods with season in DFI and DFII.

**Table 1:** Composition of the staple diet of Père David's deer in Dafeng Père David's deer National Nature Reserve, China

Season	DFI			DFII			DF III		
	Plant	RD%	Sequence	Plant	RD%	Sequence	Plant	RD%	Sequence
Summer	PAL	41.23	1	ICY	60.7	1	SAL	62.03	1
	ICY	35.86	2	PAL	20.75	2	PAU	26.04	2
	PAU	12.61	3	PAU	9.17	3	OGRP	5.88	3
	OGRP	7.97	4	OGRP	8.1	4	PAL	4.75	4
	PWT	1.55	5	DIP	0.67	5	SGL	1.1	5
	DIP	0.39	6	PWT	0.31	6	ODIP	0.21	6
	CAR	0.39	6	DSA	0.3	7			
Winter	DIP	50.87	1	DIP	52.61	1	SAL	62.01	1
	GRP	44.07	2	GRP	39.9	2	PAU	25.22	2
	PWT	5.05	3	PWT	7.49	3	ICY	8.47	3
							ODIP	3.26	4
							PAL	0.62	5
							PWT	0.23	6

Notes

CAR: Carex; DIP: Dicotyledonous plants; DSA: *Digitaria sangunalis*; EAN: *Erigeron annuus*; GRP: Gramineous plants; ICY: *Imperata cylindrica var. major*; ODIP: other dicotyledonous plants; OGRP: other gramineous plants; PAL: *Pennisetum alopecuroides*; PAU: *Phragmites australis*; PWT: Plants with trichomes; SAL: *Spartina alterniflora;* SGL: *Suaeda glauca*.

RD: percentage prevalence of each plant species in diet; sequence: foraging order; DFI, Dafeng core area 1; DFII, Dafeng core area 2; DFIII, Dafeng core area 3.

The predominant foods of the deer in DFIII were SAL (C4) and PAU (C3) in both summer and winter. There were no apparent seasonal alterations in the diets of the deer from this area. Clearly, the staple diets of the deer in DFIII differed dramatically from those of the deer from DFI and DFII, irrespective of season (Table 1). Our habitat analysis of DFIII, based on satellite images (20.01 km^2^ in 2013), indicated that SAL made up most of the plant cover (SAL: 16.49 km^2^; PAU: 1.33 km^2^; ICY: 0.26 km^2^; Figure 1c).

### 3.2 Stable isotopic differences between the feces of captive and translocated populations within the same sampling season

**Figure 2:**
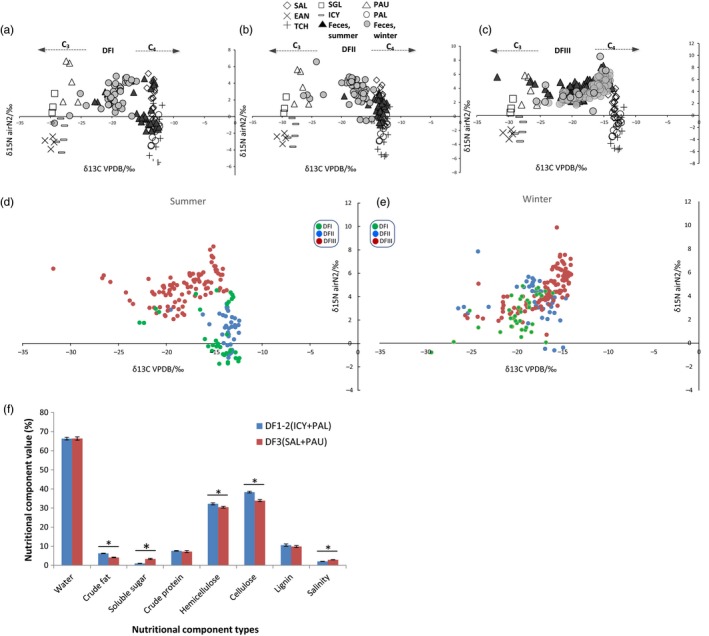
The stable carbon and nitrogen isotopes in Père David's deer habitats. (a) Isotopic changes in (b) DFI and DFII, and (c) DFIII. (d) Isotopic changes in the summer. (e) Isotopic changes in the winter. (f) The nutritional composition of the main eating plants in DFI‐II and DFIII

The stable isotope analysis also indicated that diet changed with season (especially in DFI and DFII) and that diets differed between the captive (DFI and DFII) and wild (DFIII) core areas (Figure 2). In DFI and DFII, fecal δ^13^C values in the summer were similar to those of C4 plants (Figure 2a,b). This is probably because the predominant food of deer in these two areas in the summer was C4 plants (PAL and ICY). The fecal δ^13^C values in the winter differed significantly, with summer samples generating δ^13^C values similar to those of C3 and C4 plants (Mann–Whitney *U* test, *p* < 0.05; Figure 2d; Supporting information Figure S1). Thus, mixed forage plants, including wheat (C3), soybeans (C3), and corn fibers (C4) were the bulk of the deer's diet in the winter. In DFIII, most of the fecal δ^13^C values for both summer and winter ranged between the values generated for C3 and C4 plants (Figure 2c,e). In general, the fecal δ^13^C values were closer to those of C4 plants than of C3 plants, reflecting the larger proportion of C4 plants in the deer diet: C4 (i.e., SAL), 62%; C3 (i.e., PAU), 26%. In DFIII samples collected in 2014, the differences between summer and winter values were nonsignificant (Figure S2; Supporting information Table S1), but we found the significance over summer and winter using all the samples from DFIII collecting from 2011 to 2014. These reflected the partial or shallow divergence in seasonal diets among the deer from DFIII. We observed the variation of δ^13^C between captive and translocated populations during the same season. In both summer and winter, the δ^13^C values of DFI and DFII were significantly different from those of DFIII, reflecting the differences in diet between the two regions (Supporting information Figure S1).

### 3.3 Nutritional analysis of plant food components

The nutritional composition of the main plant foods in the diets of the deer from the three core areas (DFI, DFII, and DFIII) was analyzed (Figure 2f). The main staple components of translocated Père David's deer in DFIII (SAL and PAU) had significantly lower levels of crude fat, hemicellulose, and cellulose as compared to the diets of the deer in DFI and DFII (PAL and ICY; *p* < 0.001; Figure 2f).

### 3.4 Gut microbial community dynamics in captive and translocated populations of Père David's deer

**Figure 3:**
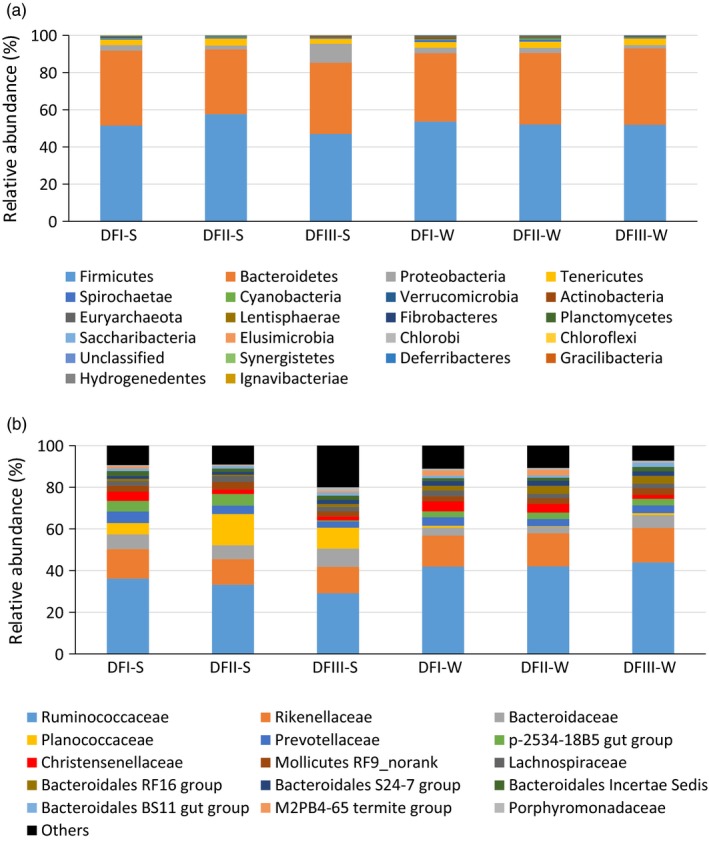
Gut microbial community dynamics in Père David's deer populations. (a) The dominant phyla. (b) The dominant families

We successfully obtained 16S rRNA gene sequences from 245 Père David's deer fecal samples (Supporting information Table S2). We chose to rarefy our sampling depth at 4,236 sequences (per sample) to equalize the sampling depth across all samples. Most of the identified microorganisms were *Firmicutes* (~52%) and *Bacteroidetes* (~38%) (Figure 3a), primarily the families *Ruminococcaceae* (~38%), *Rikenellaceae* (~14%), *Bacteroidaceae* (~6%), and *Planococcaceae* (~5%) (Figure 3b). Our Lefse analysis (with strict criteria) indicated that the *Christensenellaceae* was most significantly differently abundant among the Dafeng populations irrespective of season (as suggested by LDA score; Supporting information Figure S3). The fecal samples from the translocated population (DFIII) had a significantly lower abundance of this family (DFI: 4.6%; DFII: 3.2%; DFIII: 1.8%). The DFIII fecal samples had the lowest Shannon index irrespective of season (Figure S4).

Pairwise comparisons among core areas identified more significantly differently abundant genera between the captive and translocated populations than between the two captive populations, irrespective of season (Welch's *t* test, *p* < 0.05) (Figure S5a,b). For example, in the winter, there were 33 significantly differently abundant genera between DFI and DFII, but there were 103 significantly differently abundant genera between DFII and DFIII, and 109 significantly differently abundant genera between DFI and DFIII. This finding suggested that the gut microbial communities of the two captive populations (DFI and DFII) were more similar, and that of the translocated population (DFIII) was more dissimilar. This result was consistent with the pairwise comparisons using unweighted unifrac distance (Figure S5c,d). Irrespective of season, the pairwise comparisons between captive and translocated populations had higher dissimilarities than did the pairwise comparison between the two captive populations (Welch's *t* test, *p* < 0.05) (Figure S5c,d).

**Figure 4:**
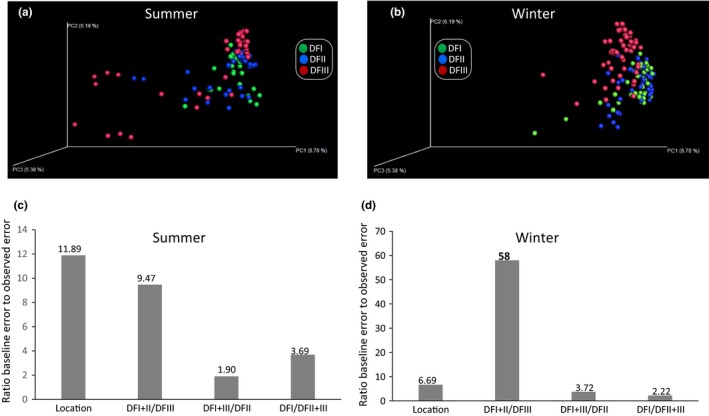
The PoCA analysis of captive and translocated populations. (a) Summer sampling season. (b) Winter sampling season. (c–d) Random forest tests for (c) the summer sampling season and (d) the winter sampling season

The PCoA showed that the translocated (DFIII) and captive populations (DFI and DFII) diverged irrespective of season (Figure 4a,b). Random forest tests were more successful differentiating captive from translocated populations than other types of classifications (e.g., DFI + DFIII and DFII, or DFI1 + DFIII and DFI; Figure 4c,d). For example, in winter‐collected fecal samples, the ratio of the baseline error to the estimated generalization error of the random forests classifier was 58 when separating DFI + DFII from DFIII. This ratio was 3.72 for differentiating DFI + DFIII from DFII and 2.22 for differentiating DFII + DFIII from DFI. This finding also indicated that the gut microbial communities of DFI and DFII were more similar than that of DFIII. One‐way PERMANOVA showed a significant difference in microbial composition among these groups (Table 2). For example, in the winter season, the pairwise comparisons detected the significant difference between translocated population (DFIII) and captive population (DFI or DFII), and no significant difference existed between DFI and DFII (Table 2). These results further confirmed the previous findings by random forest tests.

**Table 2:** The one‐way PERMANOVA test for captive and translocated population in this study using fecal bacterial species abundance (Bonferroni‐corrected *p* values)

Winter			DFII‐W	DFIII‐W	DFI‐W
Permutation *N*	9,999	DFII‐W		***0.0024***	0.9006
Total sum of squares	2.146	DFIII‐W	***0.0024***		***0.0156***
Within‐group sum of squares	2.028	DFI‐W	0.9006	***0.0156***	
*F*	4.255				
*p*	***0.0008***				
Summer					
Permutation *N*	9,999		DFII‐S	DFIII‐S	DFI‐S
Total sum of squares	5.696	DFII‐S		0.2859	0.0885
Within‐group sum of squares	5.337	DFIII‐S	0.2859		***0.0327***
*F*	3.094	DFI‐S	0.0885	***0.0327***	
*p*	***0.0103***				

The values in the pairwise comparison is the p value. The bolditalics mean the value is at significant level.

### 3.5 Changes in gut microbial function associated with their diet between captive and translocated populations

**Figure 5:**
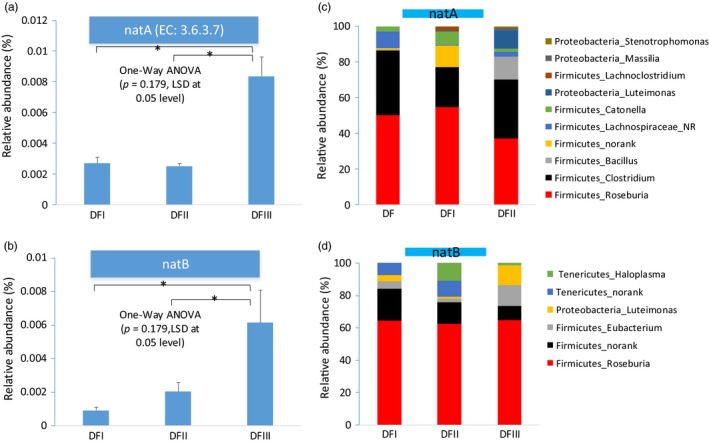
The potential adaptation on high‐salt diet by gut microbial from Père David's deer metagenomes. (a, b) The proportion of genes coding for these putative enzymes (natA and natB) related to the potential sodium transport system in Père David's deer gut microbiomes. The taxonomic assignment of the identified genes coding for natA (c) and natB (d)

Most of the primary functions identified for the gut microbes of deer from the three core areas were similar, including “carbohydrate metabolism” and “amino acid metabolism.” Considering the significant difference on the dietary nutrition between captive and translocated populations, we focus the gut microbial genes coding some putative enzymes involved in two primary functions (sodium transportation and cellulose digestion). The genes coding for two enzymes (sodium transport system ATP‐binding protein (natA) and sodium transport system permease protein (natB)) involved in the sodium transport system were enriched in the translocated Père David's deer fecal metagenomes (one‐way ANOVA, post hoc LSD test at 0.05; Figure 5a,b). Taxonomic assignment of the genes coding for natA indicated that most of them came from Firmicutes (~96%) and Proteobacteria (~4%) (Figure 5c). In genus level, most of these predicted genes came from *Roseburia* (~47%) and *Clostridium* (~30%), and others belonged to *Bacillus* (~4%), *Lachnospiraceae_norank* (~4%), and *Luteimonas* (~4%). Taxonomic assignment of the genes coding for natB revealed that most of them came from Firmicutes (~84%), Tenericutes (~10%), and Proteobacteria (~6%) (Figure 5d). In genus level, most of these genes came from *Roseburia* (~64%), *Eubacterium* (~7%), *Haloplasma* (~4%), and *Luteimonas* (6%). Thus, most of these two enzymes might come from *Roseburia*. We then investigated the relative abundance of this genus using our 16S dataset and found the relative abundance of this genus in the translocated population feces was highest (Figure S6; one‐way ANOVA test, *p* < 0.01).

**Figure 6:**
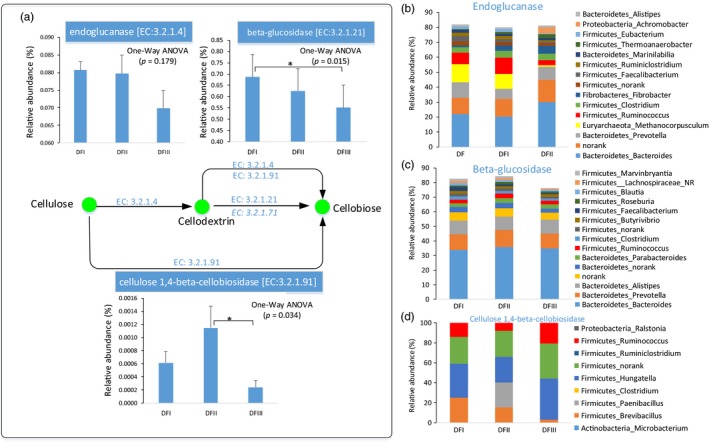
The potential for cellulose degradation by gut microbial from Père David's deer metagenomes. (a) The proportion of genes coding for these putative vital enzymes (endoglucanase, beta‐glucosidase, and cellulose 1,4‐beta‐cellobiosidase) related to the potential cellulose digestion in Père David's deer gut microbiomes. The taxonomic assignment of the identified genes coding for endoglucanase (b), beta‐glucosidase (c), and cellulose 1,4‐beta‐cellobiosidase (d)

**Figure 7:**
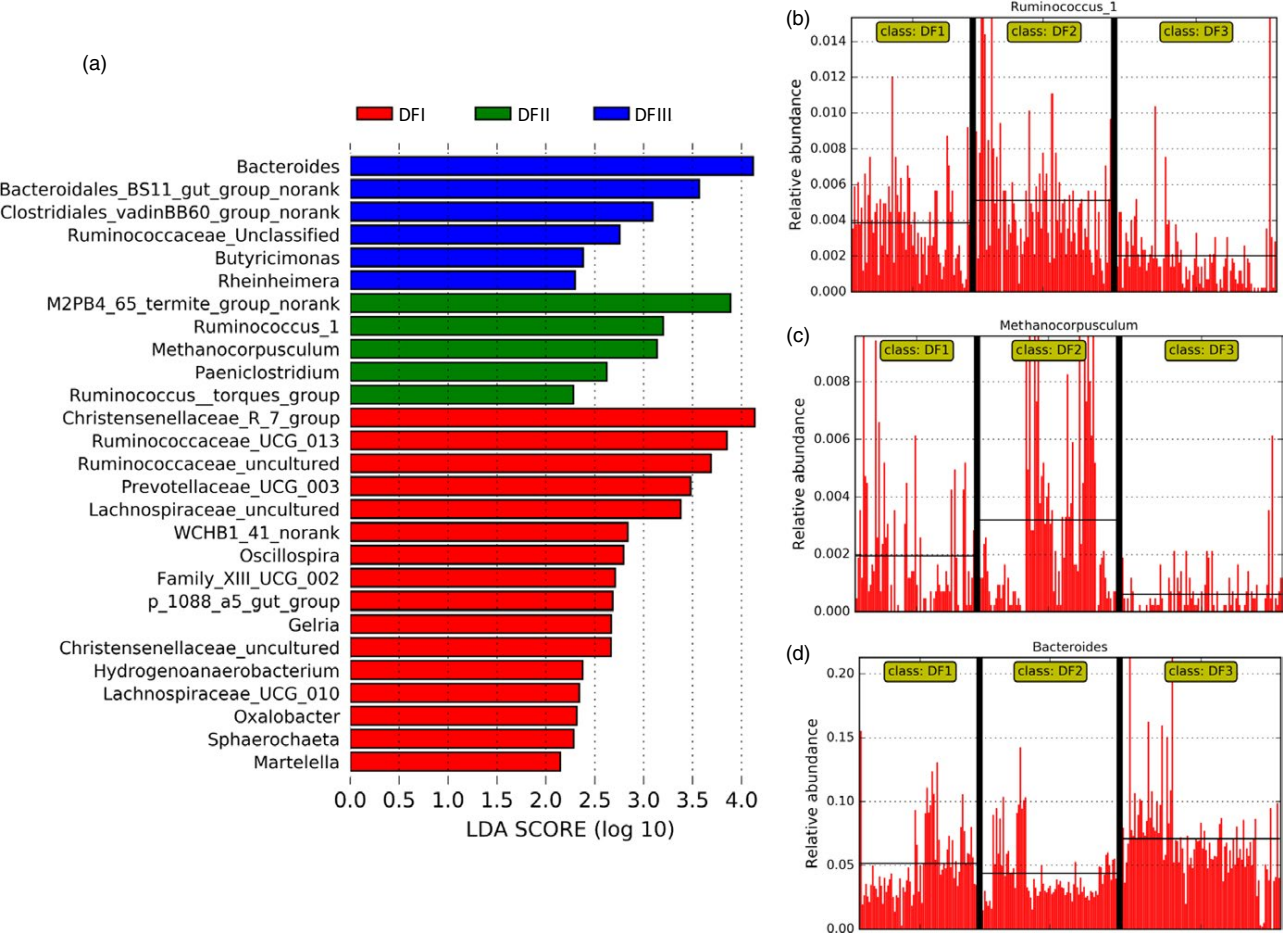
The relative abundance (16S) of some genera related to potential cellulose and sodium metabolisms in Père David's deer's feces among three populations (two captive populations: DFI and DFII, one translocated population: DFIII). (a) Linear discriminant analysis (LDA) effect size (LEfSe) method identified significant variations in the compositional profile (16S) at genus level among these populations (threshold on the logarithmic LDA score: 2.5). The relative abundance of Ruminococcus (b), Methanocorpusculum (c), and Bacteroides (d)

The proportion of putative enzymes (endoglucanase, beta‐glucosidase, and cellulose 1,4‐beta‐cellobiosidase) involved in cellulose digestion in the fecal metagenomes of the captive populations was higher than that in the fecal metagenomes of the translocated population (Figure 6a). Taxonomic classifications of these genes revealed that most of them possibly come from three phyla (Firmicutes, Bacteroidetes, and Euryarchaeota). For examples, in these main genera having these putative enzymes, *Ruminococcus* (Firmicutes) and Clostridium (Firmicutes) were the common sources for these three putative enzymes (Figure 6b,d), and the relative abundance (16S) of *Ruminococcus* was higher in captive population feces than that of translocated population feces (Figure 7a,b). The fecal gut microbiome (16S) of captive populations also has a higher proportion of *Methanocorpusculum* (Euryarchaeota) compared to that in the feces of the translocated population (Figure 7c). The feces of the translocated population had a high percentage of *Bacteroides* (Figure 7a,d). However, many other particular gut microbial genera (*Ruminococcus* and *Methanocorpusculum*) had these putative cellulose‐digestion enzymes, which might explain the relatively low proportion of these putative enzymes in the translocated population. In addition, no any predicted gene coding for putative cellulose 1,4‐beta‐cellobiosidase was detected in three of eight feces in translocation population (DFIII).

## 4 DISCUSSION

In this long‐term study (2011–2014) of the Dafeng Père David's deer captive and translocated Père David's deer populations, we observed some dissimilarities in Père David's deer gut microbiome community and function that might link microbiome composition to diet. Gut microbial communities are affected by many factors, primarily host phylogeny and diet (Ley et al., 2008). Here, the translocated deer derive from (and are genetically similar to) the captive populations. These populations have similar gut microbiome.

Ruminal cellulolytic bacteria (e.g., *Ruminococcus*) can digest the complex carbohydrates (e.g., cellulose, hemicellulose, and lignin) (Dehority & Scott, 1967; McAllister, Bae, Jones, & Cheng, 1994). The proportion of cellulolytic gut microbiome (e.g., *Ruminococcus*) and their genes coding for putative enzyme (endoglucanase, beta‐glucosidase, and cellulose 1,4‐beta‐cellobiosidase) involved in cellulose degradation in the captive populations was relatively higher than that of the translocated population. Indeed, the proportions of cellulose, hemicellulose, and lignin in the plants most consumed by the captive populations were higher than these proportions in the plants most consumed by the translocated population. Moreover, *Christensenellaceae* abundance was significantly higher in the captive populations than in the translocated population. *Christensenellaceae* has been isolated from the feces of many mammals, including humans and ruminants (Lima et al., 2015; Morotomi, Nagai, & Watanabe, 2012). Our functional analysis indicated that the *Christensenellaceae* in the Père David's deer fecal samples were mainly for “carbohydrate metabolism” and “energy metabolism” (Figure S7).

Interestingly, the abundance of *Methanocorpusculaceae* in the captive populations (~0.22%) was greater than in the translocated populations (~0.06%), especially in the winter. For example, about 11 percent in these genes coding for putative endoglucanase come from Methanocorpusculum. In the winter, the human‐provided forage most commonly straw (high fiber) and bran (Keqing, 2005). The dominant Firmicutes in the microbiota of cattle forestomachs (e.g., *Ruminococcaceae*,* Rikenellaceae*, and *Christensenellaceae*) may play essential roles in the degradation of starch and fiber (Mao, Zhang, Liu, & Zhu, 2015). Thus, the significant higher proportion of cellulose‐digestion gut microbiome (e.g., Euryarchaeota_*Methanocorpusculum*, Firmicutes_*Ruminococcus*, and Firmicutes_*Christensenella*) in captive populations might be coevolved with Père David's deer natural dietary plants and human‐provided forages.

One the other hand, one of the significant differences on dietary nutrition between captive and translocated population was the salt content. The salt concentration of the Père David's deer dietary plants in the translocation region is significantly high than that of dietary plants in captive regions. Sodium transport system plays important role in Na(+) transporting and maintaining the intra/extracellular osmotic balance in plant cell (Yamaguchi, Hamamoto, & Uozumi, 2013). In some bacteria, an Na+ circuit is an essential link between exergonic and endergonic membrane reactions (Dimroth, 1990). Here, the fecal metagenomes of the translocated population displayed the enrichment in genes coding for some putative enzymes (natA and natB) involved in sodium transport system, and most of them came from the two genera in Firmicutes, such as *Roseburia* and *Clostridium*. The relative abundance of *Roseburia* in the feces of the translocated population was higher than that in the feces of captive populations. Some *Roseburia* strains in the human gut can utilize dietary components and produce butyrate short‐chain fatty acids(Duncan, Hold, Barcenilla, Stewart, & Flint, 2002), which will affect colonic motility, immunity maintenance, and anti‐inflammatory properties (Tamanai‐Shacoori et al., 2017). Thus, we speculated that this finding on translocated Père David's deer gut microbiome might reflect some potential adaptation on host high‐salt diet and even had some putative effect on host health. In addition, this might help Père David's deer adapt to the new environment.

### 4.1 Père David's deer gut microbiome and its application in conservation management

Symbiotic gut microorganisms play an important role in host health and development (Muegge et al., 2011). In natural environments, dietary shifts are common in herbivore mammals and occur in response to food availability and plant nutritional value. Our results suggested that the difference on the dietary plant nutrition lead to some dissimilar on their gut microbial composition and function. Père David's deer gut microorganisms potentially coevolved with host diet, allowing the gut microbiome to adapt to shifts in diet in different habitats. For example, the enrichment in genes coding for some putative enzymes (natA and natB) involved in sodium transport system might help Père David's deer survive in the high‐salt diet. Currently, one issue faced by Père David's deer conservation efforts is the saturation of captive populations (Ding et al., 2014). Translocation is an effective strategy with which to address this problem. During initial Père David's deer translocation to DFIII in 1998, there was some concern about Père David's deer diet, as the high‐salt plant SAL is widely distributed across this translocation site (Ding, 2009). SAL is an invasive halophyte plant that is widely distributed in the wetlands along the Yellow Sea (Ding, 2009). Père David's deer exhibit a special behavior when feeding on SAL: The deer repeatedly feed on the same plant over a long period, causing the plant to continue to grow fresh leaves (Ding, 2009). In the nearly 20 years since the initial translocation, the Père David's deer population at DFIII has grown well (Ding, 2017). Given that the evolutionary signature gut microbiome of the translocated populations, the large area of SAL*‐*dominant wetlands along the Yellow Sea might be the main region for future Père David's deer translocations. Thus, here, we provide the importance of gut microbiome of the translocation population on the potential adaptation to the new environment (e.g., diet), which can be applied and relevant for the efficiency conservation management.

## CONFLICT OF INTERESTS

The authors declare that there is no conflict of interest.

## AUTHOR CONTRIBUTIONS

LZ designed the study. LW, JD, ZY, R.Y, and YD performed the experiments. Q.D carried out the analysis on satellite maps. LZ and HC performed analyses. LZ wrote the manuscript with comments from all co‐authors.

## Data Availability

DNA sequences have been deposited in figshare (https://doi.org/10.6084/m9.figshare.7388777.v1).
