# Supplementary material for: Père David’s deer gut microbiome changes across captive and translocated populations: Implications for conservation
Source: Evol Appl. 2019 Jan 3;12(3):622–35. doi: 10.1111/eva.12743 (PMC6383733; doi:10.1111/eva.12743)
Supplement: Supplementary file 1 [file EVA-12-622-s001.docx]

**SUPPORTING INFORMATION**

Table S1 The test onδ13C values of each group

| Total | DF1-S | DF1-W | DF2-S | DF2-W | DF3-S | DF3-W |  |
| --- | --- | --- | --- | --- | --- | --- | --- |
| DF1-S |  |  |  |  |  |  |  |
| DF1-W | 0.000 |  |  |  |  |  |  |
| DF2-S | NS | 0.000 |  |  |  |  |  |
| DF2-W | 0.000 | NS | 0.000 |  |  |  |  |
| DF3-S | 0.000 | 0.052 | 0.000 | NS |  |  |  |
| DF3-W | 0.001 | 0.000 | 0.000 | NS | 0.014 |  |  |
| 2014 | DF1-S | DF1-W | DF2-S | DF2-W | DF3-S | DF3-W |  |
| DF1-S |  |  |  |  |  |  |  |
| DF1-W | 0.000 |  |  |  |  |  |  |
| DF2-S | NS | 0.000 |  |  |  |  |  |
| DF2-W | NS | NS | 0.005 |  |  |  |  |
| DF3-S | 0.006 | NS | 0.000 | NS |  |  |  |
| DF3-W | NS | NS | 0.000 | NS | NS |  |  |

NS, non-significant. The values in the table were the *p* values.

Table S2 The sample period and sample sizes (fecal samples) used in this study

| **Isotopic** | DF1 | DF2 | DF3 |  |
| --- | --- | --- | --- | --- |
| 2011-2012 winter | 4 | 9 | 66 |  |
| 2012-summer |  |  | 43 |  |
| 2013-summer | 8 | 10 | 20 |  |
| 2014-summer | 26 | 26 | 32 |  |
| 2014-winter | 36 | 39 | 47 |  |
| **Gut microbes Miseq** | DF1 | DF2 | DF3 |  |
| 2011-2012 winter | 4 | 11 | 10 |  |
| 2013-summer | 5 | 9 | 10 |  |
| 2014-summer | 25 | 20 | 26 |  |
| 2014-winter | 36 | 41 | 48 |  |
| **Gut microbes Metagenomics*** | DF1 | DF2 | DF3 |  |
| 2014-summer | 4 | 4 | 4 |  |
| 2014-winter | 4 | 4 | 4 |  |

*The samples in gut microbes Miseq included these 30 samples.

Figure S1 The mean stable carbon values in summer and winter across all samples collected between 2011 and 2014. The statistic tests were done using Mann–Whitney U test. *, *p* < 0.05; **, *p* < 0.01, ***, *p* < 0.001. The numbers inside the bars mean the sample size of feces. . The calculation of this formula ( *δ*^13^C = [(^13^C/^12^C)_sample_ / (^13^C/^12^C)_standard_ - 1] × 1000) gained the y values. The *δ*^13^C of C3 and C4 plants normally are non-overlapping. Thus, the y value can reflect the types of the dietary plants.

c

Figure S2 The mean stable carbon values in the different seasons using the total number of samples, collected in 2014. The statistic tests were done using Mann–Whitney U test. *, *p* < 0.05; **, *p* < 0.01, ***, *p* < 0.001. The numbers inside the bars mean the sample size of feces. The calculation of this formula ( *δ*^13^C = [(^13^C/^12^C)_sample_ / (^13^C/^12^C)_standard_ - 1] × 1000) gained the y values. The *δ*^13^C of C3 and C4 plants normally are non-overlapping. Thus, the y value can reflect the types of the dietary plants.

Figure S3 Lefse analysis of gut microbial composition in the summer sampling season and winter sampling season.


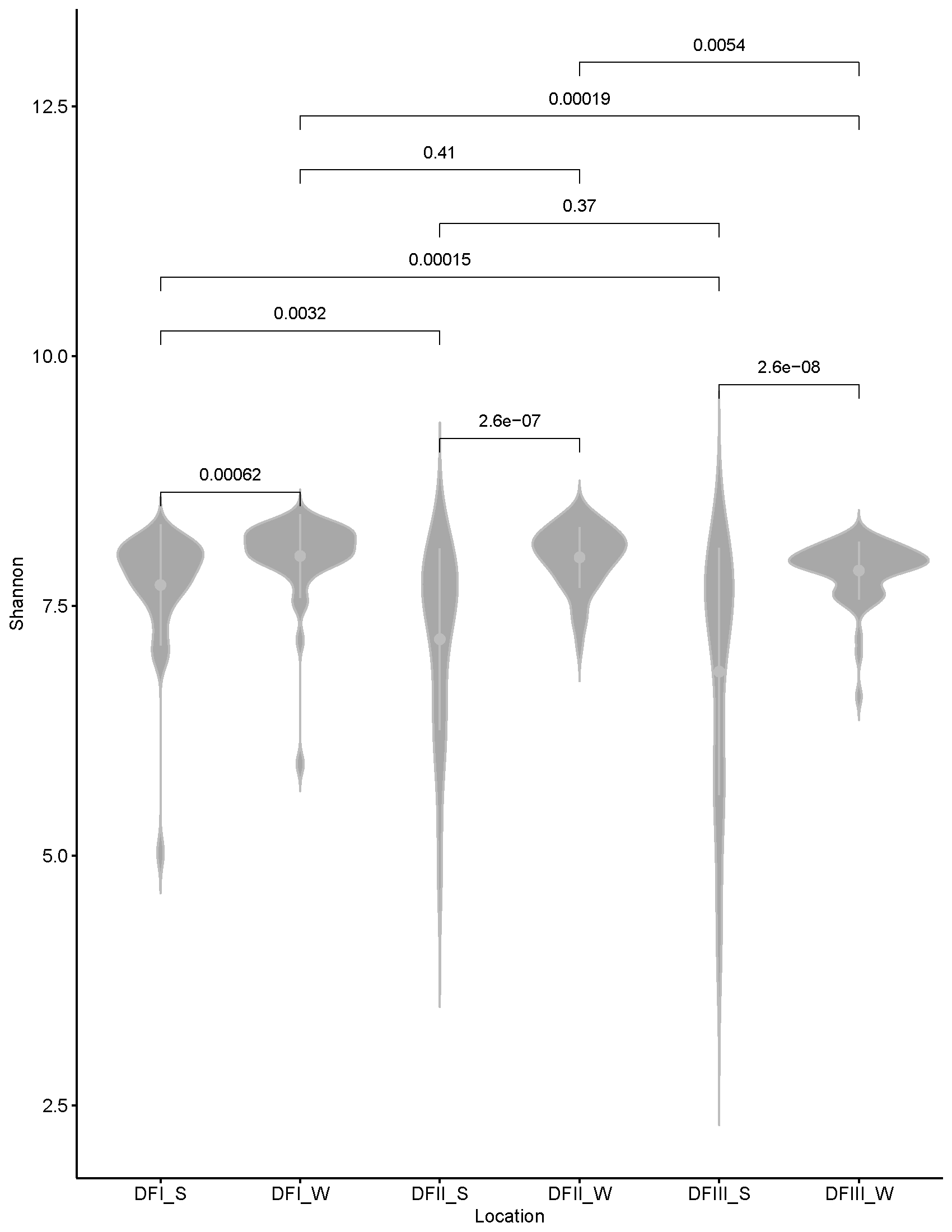


Figure S4 The Shannon index among these groups.

Figure S5. The dissimilarities between the fecal gut microbiome of the captive and translocated populations. (A) The number of genera that differed significantly in abundance between pairs of populations in the summer sampling season. (B) The number of genera that differed significantly in abundance between pairs of populations in the winter sampling season. (C) The pairwise unweighted unifrac distances in the summer sampling season. (D) The pairwise unweighted unifrac distances in the winter sampling season.


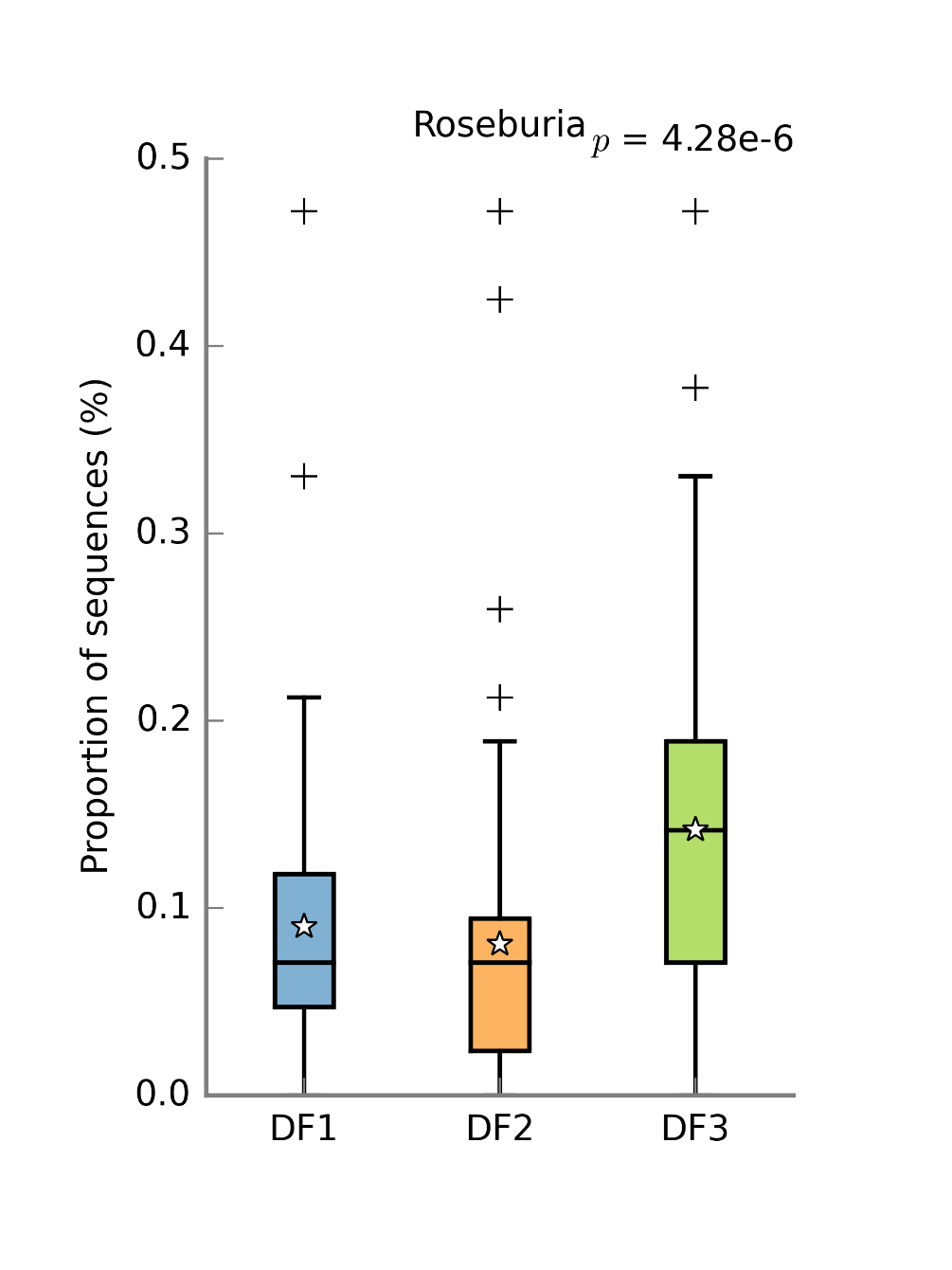


Figure S6 The relative abundance of *Roseburia* in the feces among three populations

Figure S7 The putative function of Christensenellaceae in Milu fecal metagenomes
